# 5d, a novel analogue of 3-n-butylphthalide, decreases NADPH oxidase activity through the positive regulation of CK2 after ischemia/reperfusion injury

**DOI:** 10.18632/oncotarget.8548

**Published:** 2016-05-12

**Authors:** Jia Zhou, Yi-hua Zhang, Hui-zhu Song, Hui Ji, Xiao-li Wang, Lei Wang, Jun Qian, Jing-jing Ling, Feng-feng Ping

**Affiliations:** ^1^ School of Pharmaceutical Science, Jiangnan University, Wuxi, P.R. China; ^2^ State Key Laboratory of Natural Medicines, Center of Drug discovery, China Pharmaceutical University, Nanjing, P.R. China; ^3^ Wuxi People's Hospital affiliated to Nanjing Medical University, Wuxi, P.R. China

**Keywords:** 5d, neuroprotection, CK2, NADPH oxidase, ROS, Neuroscience

## Abstract

5d, a novel analogue of the racemic 3-*n*-butylphthalide (NBP), has been reported for its free radical scavenging activity *in vitro* and preventive neuroprotection *in vivo*. Nevertheless, the mechanism by which 5d attenuated ischemia/reperfusion (I/R) injury is still unknown. Our results showed that 5d significantly increased CK2 activity as well as CK2α and 2α' protein levels after I/R injury. Besides, 5d suppressed the translocation of cytosolic p47phox and Rac1 to the membrane, decreased NOX4 expression and ROS generation. Furthermore, 5d blocked the dissociation between CK2α and Rac1 so as to decrease NADPH oxidase activity. Based on these findings, we propose that the neuroprotective effect of 5d is due to an increase of CK2 activity, which blocks I/R-induced dissociation between CK2α and Rac1, decreases NADPH oxidase activity, inhibits ROS production and finally realizes the neuroprotection of I/R. These findings point to that 5d might be considered an attractive candidate for further studies in ischemic stroke.

## INTRODUCTION

Strokes are the leading cause of permanent disability and death worldwide. Due to the occlusion of a vessel in the brain, ischemic strokes account for the majority of strokes [1, 2]. After brain ischemia, the increased oxidative stress has been considered as a primary cause for brain injury due to oxidation of proteins and nucleic acids, and peroxidation of lipid induced by free radical [3, 4]. The application of free radical scavengers as potential treatments for ischemic stroke has attracted more and more attention [5, 6].

3-*n*-butylphthalide (NBP) has been approved by State Food and Drug Administration (SFDA) of China as a new drug for treating ischemic stroke [7]. Using NBP as the parent compound, we designed 3-butylbenzo[*c*]thiophen-1(3*H*)-one, named 5d, a new analogue of the racemic NBP. To improve its therapeutic effect, NBP is often administered with anti-platelet and/or antioxidant drug(s) [8]. Interestingly, it has been recently reported the actions of 5d against free radicals, platelet aggregation, thrombosis and cerebral ischemia [9]. Nevertheless, the mechanism of 5d against cerebral ischemia is still unknown.

NADPH oxidases are known to be the only enzyme family producing reactive oxygen species (ROS) as their sole and primary function [10, 11]. As the multi-protein complexes, they are comprised of a catalytic, transmembrane-spanning subunit (NOX) and several structure and regulatory proteins which localized in both the cytosol and the membrane [12]. To activate NADPH oxidase, cytosolic subunits guanosine 5′-triphosphate (GTP)-Rac1, p67phox and p47phox migrate from cytoplasm to the membrane forming the active NADPH oxidase complex [13, 14]. It has been reported that protein kinase CK2 protects against cerebral ischemia through the negative regulation of NADPH oxidase [15]. CK2 is a highly conserved protein kinase. It has a growing list of more than 300 substrates and the majority are proteins implicated in nuclear functions such as ubiquitination, cell survival, gene expression, signal transduction, and RNA synthesis [16, 17]. In recent years, the study on the role of CK2 in cerebral ischemia has received more and more attention of researchers [15, 18, 19].

In the present experiment, the middle cerebral artery occlusion (MCAO) was used as an *in vivo* model, while the oxygen-glucose deprivation/reoxygen (OGD/R) was used as an *in vitro* model. The aim of this text is to investigate whether CK2 and/or NADPH oxidase are involved in neuroprotection of 5d against the cerebral ischemia and its relevant mechanism.

## RESULTS

### 5d elevated CK2 activity in an *in vivo* MCAO model and *in vitro* OGD/R model

**Figure 1:**
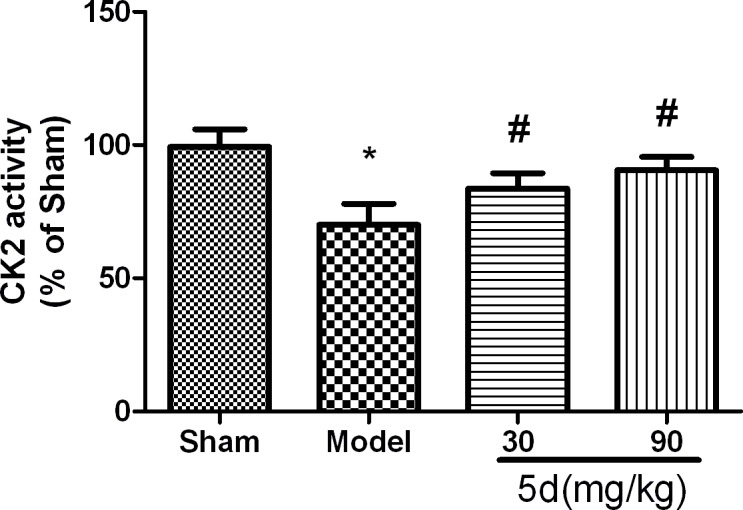
Effects of 5d on CK2 activity in the brains of rats after I/R Data are expressed as means ± SD (*n* = 6). ^*^P < 0.05 *vs* Sham group. ^#^*P* < 0.05 *vs* Model group.

**Figure 2:**
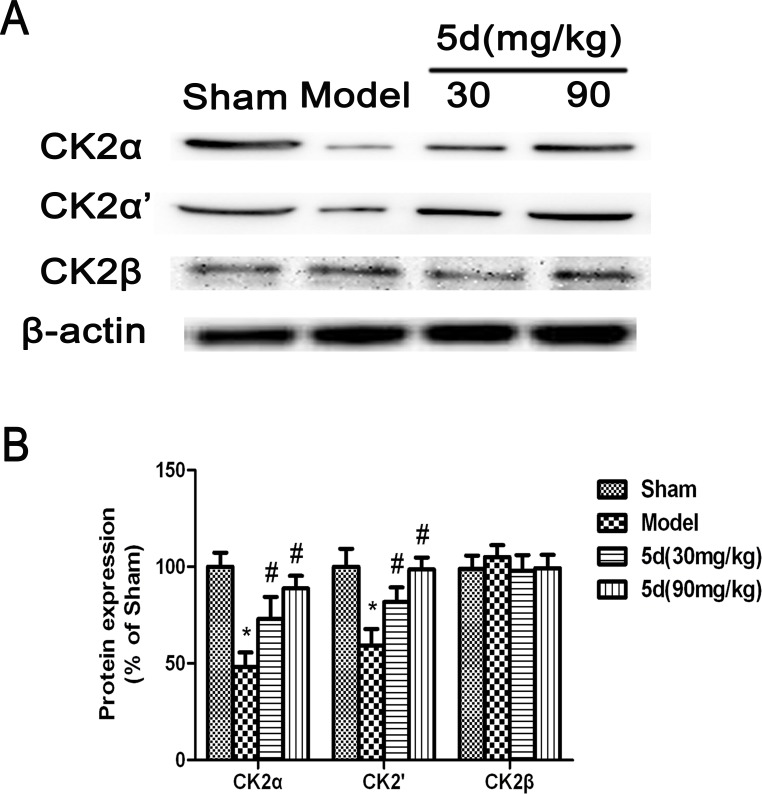
Effects of 5d on CK2α, CK2α'and CK2β protein levels in the brains of rats after I/R **A.** Representative Western blots of CK2α, CK2α'and CK2β protein levels in the brains of rats. **B.** CK2α, CK2α'and CK2β proteins expression were normalized to b-actin level. Data are expressed as means ± SD (*n* = 4). ^*^P < 0.05 *vs* Sham group. ^#^*P* < 0.05 *vs* Model group.

As shown in Figure 1, compared with the Sham groups, CK2 activity was remarkably reduced in the brains of Model groups. 5d elevated CK2 activity after I/R. To reveal which subunit was involved in 5d-induced increase of CK2 activity, we then assessed the levels of CK2α, 2α' and 2β protein expression. The levels of CK2α and 2α' protein expression were strongly decreased in the brains of Model groups verses that of Shams. 5d significantly up-regulated CK2α and 2α' protein expression after I/R injury. However, the expression of CK2β protein between groups did not show any differences (Figure 2).

**Figure 3:**
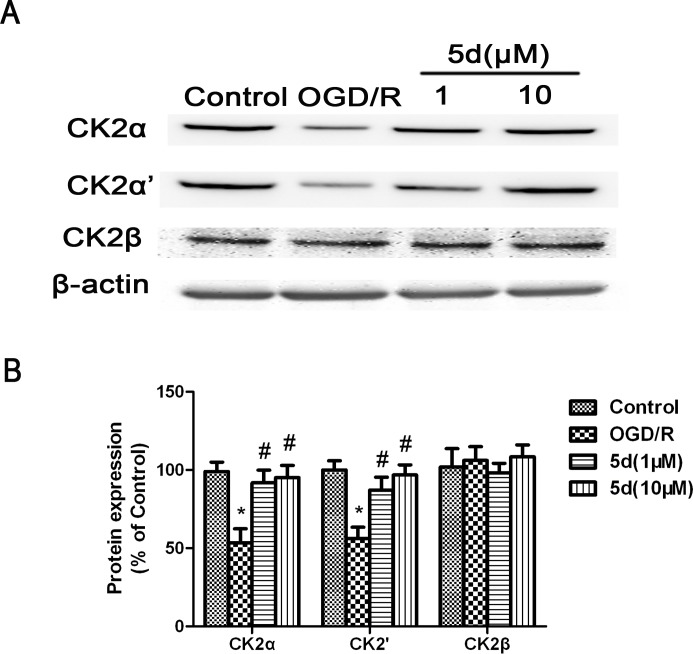
Effects of 5d on CK2α, CK2α'and CK2β protein levels in cortical neurons after OGD/R **A.** Representative Western blots of CK2α, CK2α'and CK2β protein levels in cortical neurons. **B.** CK2α, CK2α'and CK2β proteins expression were normalized to b-actin level. Data are expressed as means ± SD (*n* = 4). ^*^P < 0.05 *vs* Control group. ^#^*P* < 0.05 *vs* OGD/R group.

To confirm the changes of CK2 subunits protein expression, the primary cortical neuronal culture was subjected to OGD/R injury. As with the *in vivo* data, pretreatment with 5d profoundly attenuated the down-regulation of CK2α and 2α' protein expression induced by OGD/R, whereas there was no influence on the levels of CK2β protein expression (Figure 3).

### 5d decreased the level of NOX4 protein expression after I/R

**Figure 4:**
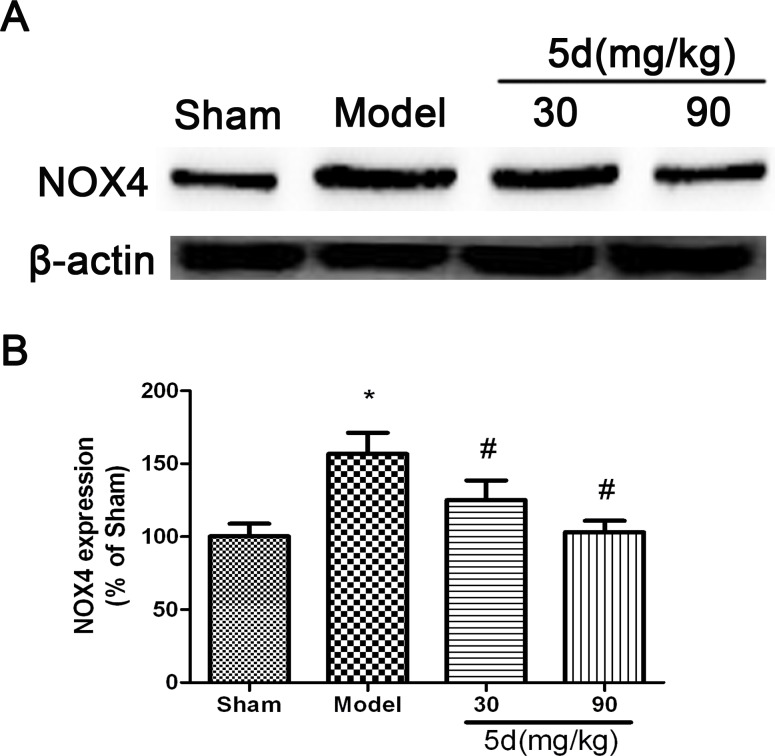
Effects of 5d on NOX4 protein level in the brains of rats after I/R **A.** Representative Western blots of NOX4 protein level in the brains of rats. **B.** NOX4 protein expression were normalized to b-actin level. Data are expressed as means ± SD (*n* = 4). ^*^P < 0.05 *vs* Sham group. ^#^*P* < 0.05 *vs* Model group.

NADPH oxidases are known to be the only enzyme family with the sole function of producing ROS. NOX4, as the most widely distributed isoform of the catalytic NADPH oxidase subunits (NOX), has been demonstrated to be a promising therapeutic target for diseases including stroke, fibrosis, and heart failure. Accordingly, in the current study, we detected the level of NOX4 protein expression in the brains of rats. Results showed that the level of NOX4 expression was up-regulated in the brains of Model group compared with that of Shams. 5d strongly down-regulated the level of NOX4 expression after I/R injury (Figure 4).

**Figure 5:**
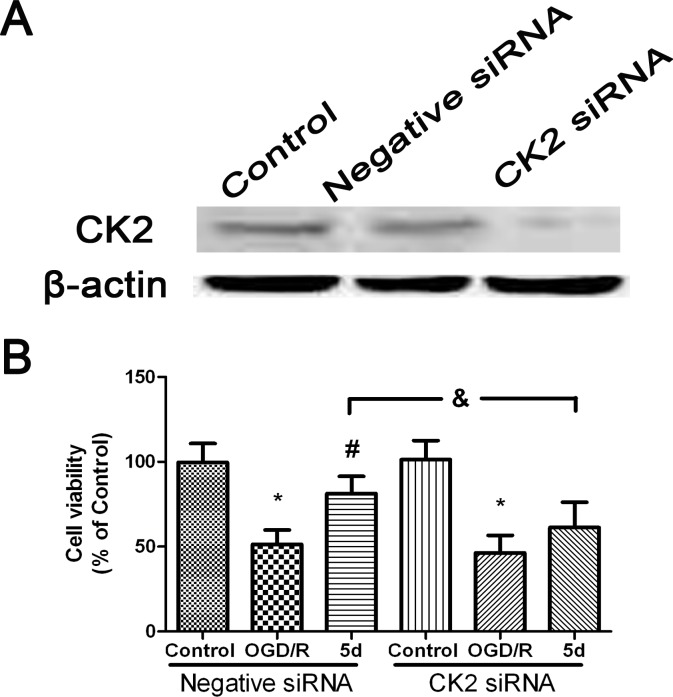
5d increased the neuronal viability through up-regulation of CK2 expression **A.** Cortical neurons were transfected with Negative siRNA or CK2 siRNA for 24 h, and then cells were harvested to detect CK2 protein expression by Western blot. **B.** Cortical neurons were transfected with siCK2 or Negative siRNA for 24 h. The transfected cells were treated with 5d for 24 h before OGD/R treatment. Cell viability was measured by MTT assay. Data are expressed as means ± SD (*n* = 4). ^*^P < 0.05 *vs*. Control group, ^#^*P* < 0.05 *vs*. OGD/R group.

**Figure 6:**
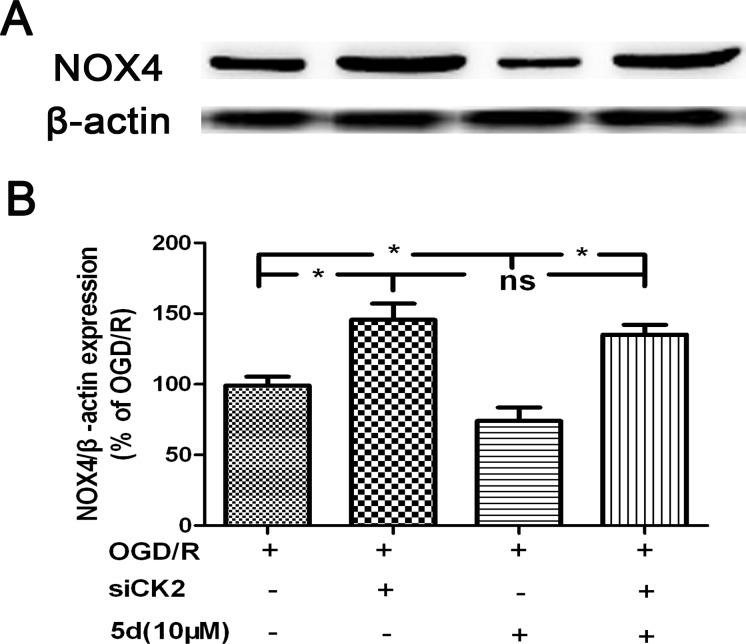
CK2 negatively modulates the expression of NOX4 protein **A.** Cortical neurons were transfected with siCK2 for 24 h. The transfected cells were treated with 5d for 24 h before OGD/R and the expression of NOX4 protein was examined by Western blot. **B.** Statistical results from the densitometric measurements after normalization against b-actin were calculated as the means ± SD (*n* = 4). Values are expressed as a percentage of the corresponding OGD/R value. ^*^p < 0.05; ns, nonsignificant.

To define the role of CK2 in 5d-induced down-regulation of NOX4 expression after I/R, a primary neuronal culture was subjected to OGD/R with the transfection of CK2α-specific siRNA before 5d treatment. Figure 5A showed that in comparison with untransfected or scrambled-transfected primary neuronal cells, the level of CK2α protein expression was significantly decreased by 24 h with the transfection of CK2α siRNA. Besides, CK2 down-regulation remarkably reduced neuronal viability in response to OGD/R injury. It is worthy to mention that the improvement of 5d on neuronal survival was markedly curtailed in CK2siRNA-transfected neurons (Figure 5B). Moreover, blocking CK2α protein expression in cortical primary neurons impaired the effect of 5d on decreasing the level of NOX4 protein expression after OGD/R injury (Figure 6).

### 5d blocked the translocation of p47phox and Rac1 from cytoplasm to membrane

**Figure 7:**
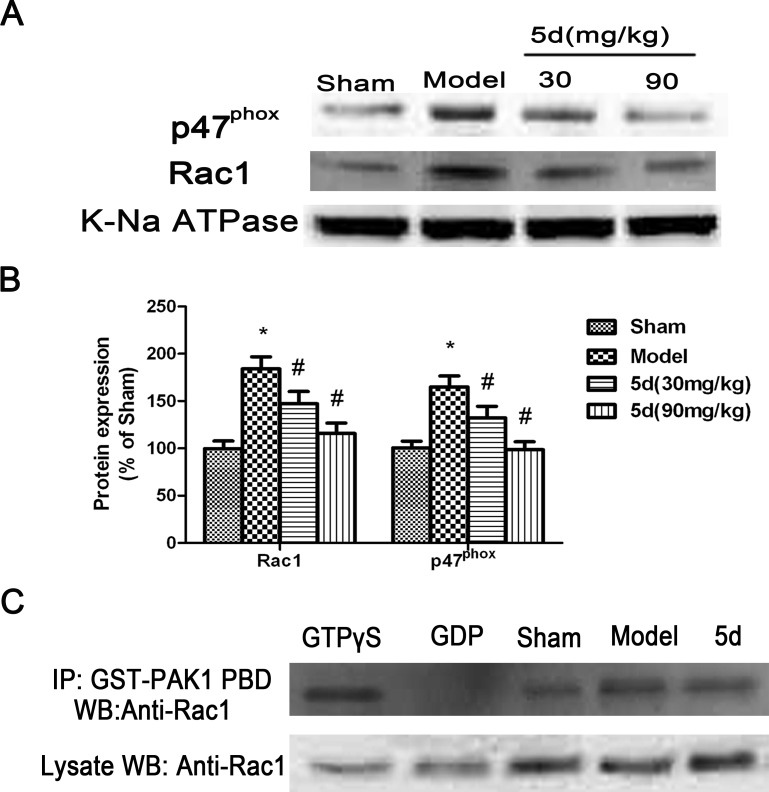
Effects of 5d on the translocation of cytosolic p47phox and Rac1 to membrane **A.** Representative Western blots of p47phox and Rac1 protein expression in membrane fractions from the brains of rats. K-Na ATPase antibody was applied as a membrane marker. **B.** The expression of p47phox and Rac1 protein in the membrane were normalized to K-Na ATPase level. **C.** Rac1 activity was measured by co-IP using GST-PAK1 PBD, followed by Western blots with a Rac1 antibody (*n* = 6 per group). Data are expressed as means ± SD (*n* = 4). ^*^P < 0.05 *vs* Sham group. ^#^*P* < 0.05 *vs* Model group.

It is known that NADPH oxidase activation can be initiated when the cytosolic subunits GTP-Rac1, p67phox, and p47phox are translocated to the membrane to form the NADPH oxidase complex. Therefore, to detect the degree of translocation of p47phox, p67phox, and Rac1, brain tissues of rats were separated into cytosolic and membrane fractions and then assayed by Western blot with these subunits' antibodies. As shown in Figure 7A and 7B, the levels of Rac1 and p47phox protein expression were profoundly decreased in the membrane fractions from 5d-treated brains compared with that from vehicle-treated brains under I/R conditions. Besides, by immunoprecipitation with the GST-PAK1 PBD beads, 5d induced an obvious decrease in Rac1 activity under I/R conditions compared with the Sham group (Figure 7C). The results above indicated that 5d prevented the translocation of subunits from cytoplasm to the membrane after I/R. However, the levels of p67phox protein expression in the membrane fractions was not obviously changed after I/R (data not shown).

### 5d inhibited the dissociation of CK2α and Rac1

**Figure 8:**
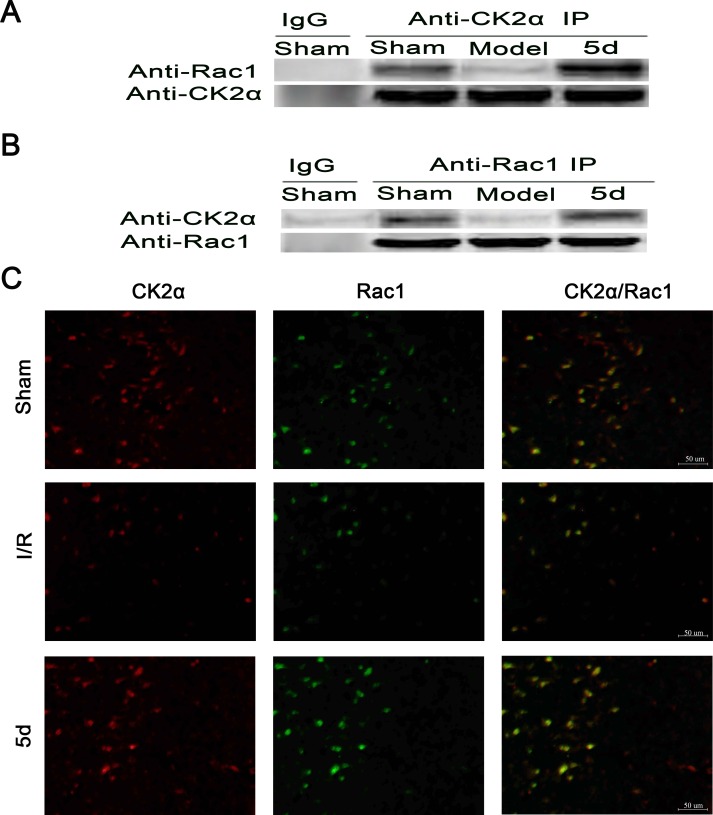
Effects of 5d on the relationship between CK2 and Rac1 protein levels Brain tissue lysates of the Sham or Mode group were immunoprecipitated with an anti-CK2α antibody **A.** or an anti-Rac1 antibody **B.** followed by Western blots with an anti-Rac1 antibody and an anti-CK2α antibody (*n* = 4). **C.** Double labeling with an anti-CK2α antibody and an anti-Rac1 antibody using tissue lysates from brains of the Sham or Mode group (*n* = 6 per group).

To explore how the loss of CK2 activity induced by I/R promotes the activation of NADPH oxidase *via* translocation of Rac1 and p47phox in cerebral ischemia, we examined the possibly physical interactions between CK2α and p47phox or between CK2α and Rac1. Figure 8A and 8B showed that there existed an interaction between CK2α and Rac1 under normal conditions, which was disrupted after I/R injury. 5d blocked the dissociation of CK2α and Rac1 after I/R. To verify the specificity of the CK2α antibody applied in the co-IP assay, the samples of Sham group was immunoprecipitated with normal IgG as a negative control. The results showed that there were no interacting bands between CK2α and Rac1. Furthermore, to confirm the direct interaction between CK2α and Rac1, an immunofluorescent method was applied. As shown in Figure 8B, CK2α immunopositive cells in the brains of Sham group were mainly colocalized with the Rac1 immunopositive cells, whereas the coimmunoreactive cells were obviously diminished under I/R conditions (Figure 8B). These data indicate that after I/R injury, the direct interaction between Rac1 and CK2α is broken, Rac1 releases from CK2α and translocates to membrane. Our results found that 5d treatment reversed this process by protecting the interaction between CK2α and Rac1 from damage induced by I/R, and blocking the translocation of Rac1 to the membrane.

### 5d suppressed ROS production through up-regulation of CK2 activity after I/R

**Figure 9:**
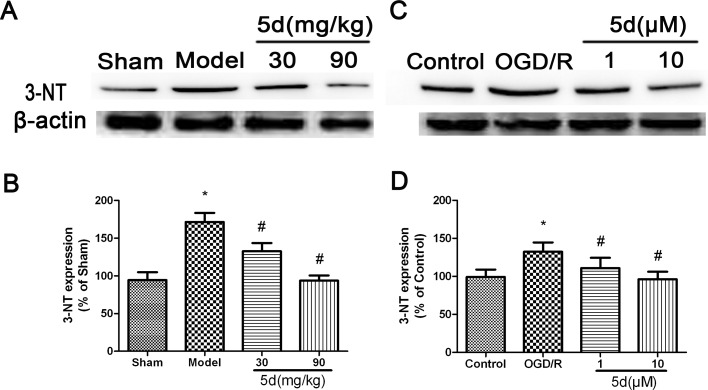
Effects of 5d on 3-NT protein expression in the brains of rats after I/R and in cortical neurons after OGD/R **A.** Representative Western blots of 3-NT protein expression in the brains of rats. **B.** 3-NT protein expression in the brains of rats were normalized to b-actin level. **C.** Representative Western blots of 3-NT protein expression in cortical neurons after OGD/R. **D.** 3-NT protein expression in cortical neurons were normalized to b-actin level. Data are expressed as means ± SD (*n* = 4). ^*^P < 0.05 *vs* Sham group or Control group. ^#^*P* < 0.05 *vs* Model group or OGD/R group.

**Figure 10:**
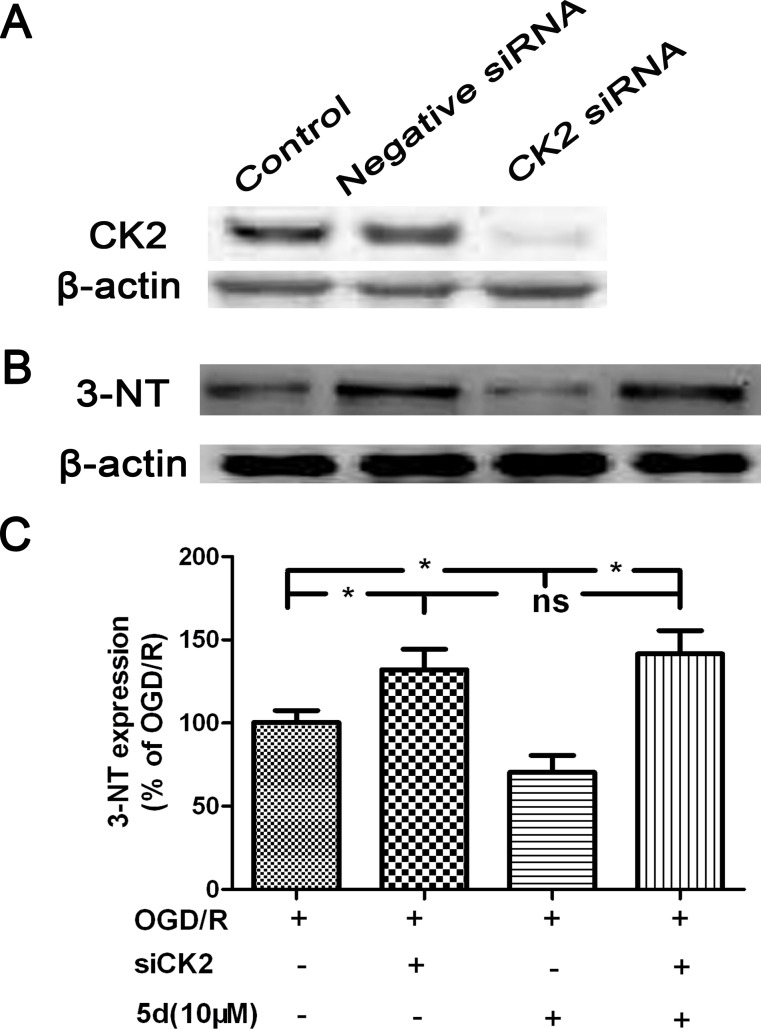
CK2 negatively modulate the expression of 3-NT protein **A.** Cortical neurons were transfected with Negative siRNA or CK2 siRNA for 24 h, and then cells were harvested to detect CK2 protein expression by Western blots. **B.** Cortical neurons were transfected with siCK2 for 24 h. The transfected cells were treated with 5d for 24 h before OGD/R and the expression of 3-NT protein was examined by Western blot. **C.** Statistical results from the densitometric measurements after normalization against b-actin were calculated as the means ± SD (*n* = 4). Values are expressed as a percentage of the corresponding OGD/R value. ^*^P < 0.05; ns, nonsignificant.

In order to quantify the levels of ROS production, we evaluated the expression of 3-nitrotyrosine (3-NT) protein by Western blot *in vivo* and *in vitro*. As shown in Figure 9A and 9B, in comparison with the vehicle-treated brains, the level of 3-NT protein expression was markedly enhanced in the brains of Model group. 5d notably decreased 3-NT protein expression after I/R injury. In consistence with the *in vivo* results, *in vitro* experiment indicated that 5d profoundly suppressed the elevation of 3-NT protein expression induced by I/R injury (Figure 9C and 9D). Furthermore, in comparison with scrambled-siRNA-transfected or untransfected samples, CK2α-specific siRNA transfection for 48 h in the cortical neurons after OGD/R prominently abolished 5d-induced down-regulation of 3-NT protein expression (Figure 10). These data suggest that 5d inhibits ROS production through elevating CK2 activity during I/R.

## DISCUSSION

Ischemic stroke has been demonstrated to be triggered by an imbalance of antioxidant defense system and oxidative stress. Thus, use of antioxidants might possibly limit oxidative damage so as to ameliorate disease progression [20–22]. We recently reported the free radical scavenging potential of 5d, coupled with the neuroprotective effect on cerebral ischemia [9]. However, further study is required to elucidate the possible mechanism(s) of 5d against ischemic stroke. In the present study, we provided evidence demonstrating that after I/R injury, 5d decreased NADPH oxidase activity through the positive regulation of CK2.

Protein kinase CK2 is a serine/threonine kinase comprised of α, α' and β, of which α and α' are catalytic subunits, and β is a regulatory subunit [23, 24]. There is literature demonstrating the neuroprotection of CK2 after ischemia stroke [15, 18, 19]. In line with previous studies, our results showed that CK2 activity together with CK2α and 2α' protein expression were significantly down-regulated after I/R injury. Interestingly, 5d treatment obviously increased CK2 activity as well as CK2α and 2α' protein levels after I/R.

The NADPH oxidase family of enzymes, also called NOXs, produce ROS as their primary function. Consequently, their roles in tightly regulated redox signaling pathways are areas of intense study [25, 26]. The activation of NADPH oxidase is initiated by translocation of Rac1, p67phox, and p47phox from cytoplasm to the membrane [13, 27]. In an HL-60 cell line, Park and his co-workers found that CK2 can phosphorylate p47phox, as well as CK2 inhibition by DRB can facilitate translocation of p47phox [28]. Along with these lines, we demonstrated in our study that 5d-induced increase of CK2 activity blocked the translocation of cytosolic Rac1 and p47phox to the membrane. Meanwhile, the level of NOX4 protein expression, the most widely distributed isoform of NADPH oxidase subunits (NOXs), was obviously declined in the brains of 5d-treated rats. However, CK2-specific siRNA impaired 5d's inhibitory effect on NOX4 protein expression. Of note, we found that I/R injury promoted the dissociation between CK2α and Rac1, which could be suppressed after 5d administration. These findings indicate that 5d could prevent the dissociation between CK2α and Rac1 so as to block NADPH oxidase activation after I/R.

In the present study, it was also demonstrated that 5d strongly depressed I/R- or OGD/R-induced ROS generation evidenced by the down-regulation of 3-NT protein expression. Blocking CK2α protein expression by CK2 siRNA in primary cortical neurons weakened the inhibitory effect of 5d on 3-NT protein expression. Thus, we suggest that 5d exhibits neuroprotective effect against the I/R or OGD/R injury *via* inhibiting ROS generation induced by NADPH oxidase activation through the elevation of CK2 activity.

Taken together, our data revealed the mechanism underlying 5d's neuroprotection on cerebral ischemia. This occurs *via* an increase of CK2 activity, which obstructs I/R-induced dissociation between CK2α and Rac1, decreases NADPH oxidase activtiy, inhibits ROS production and finally realizes the protective effect of I/R. Despite the current results, further research is needed to exploit the remaining potential of 5d in cerebral ischemia. Following studies in our laboratory will focus on whether 5d could induce a late phase neuroprotection, diffuse across biological membrane and blood-brain barrier, affect mitochondrial function as well as the relevant mechanisms involved.

## MATERIALS AND METHODS

### Chemicals and reagents

5d, which purity > 99%, was synthesized by the Center of Drug Discovery, China Pharmaceutical University. Its molecular structure was shown in previous studies [9, 29] and was dissolved in normal saline. Membrane and cytosol protein extraction kit and Lactate dehydrogenase (LDH) kit were obtained from Beyotime Institute of Biotechnology (Haimen, China). Rac1 activation assay kit was purchased from Millipore Corporation (MA, USA). The primary antibodies used were polyclonal antibodies against CK2α, CK2α', CK2β, NOX4, Rac1, p47^phox^, p67^phox^, β-actin (Millipore Corporation, MA, USA). The goat anti-rabbit IgG-HRP secondary antibody was supplied by Bioworld Technology Inc. (MN, USA). NBP, MTT (3-(4,5-Dimethylthiazol-2-yl)-2,5-diphenyltetrazolium bromide), and all other chemicals were purchased from Sigma Chemical Co., (St. Louis, MO, USA).

### Animals

Adult male Sprague-Dawley rats (SD) weighing 250~300 g were purchased from B&K Universal Group Limited (Shanghai, China). The rats were kept under standard laboratory conditions with light from 08:00 a.m. to 08:00 p.m. Animals were free access to food and water. Animals care and treatment in this study observed the National Institute of Health Guidelines for the Care and Use of Laboratory Animals. All procedures were conducted in accordance with the Institutional Animal Care and Utilization Committee of China Pharmaceutical University.

### Middle cerebral artery occlusion (MCAO) and drug administration

After anesthetized i.p. with 350 mg/kg chloral hydrate (Sinopharm Chemical Reagent, Beijing, China), all rats were subjected to the middle cerebral artery occlusion (MCAO), a procedure described previously [9]. During the experiment, the cerebral blood flow (CBF) was monitored by laser Doppler flowmetry (LDF 100C, BIOPAC Systems) to ensure that occlusion of the MCA with the specific suture resulted in >80% decline in CBF [30]. The control group received a sham surgery. The core body temperature of individual animals was monitored with a rectal probe and maintained at 37 ± 0.5°C during the whole procedure.

All rats were divided randomly into four groups as following: (1) Sham group, (2) Model group, (3) I/R + 5d (30 mg/kg, i.g.) group, (4) I/R + 5d (90 mg/kg, i.g.) group. The compound of 5d was administered daily, starting at the onset of reperfusion and continued for 3 days.

### Coimmunoprecipitation (co-IP) assay

At 3 d after reperfusion, brains were isolated and lysed on ice using lysis buffer, which contains 25 mM HEPES (pH 7.7), 0.4 M NaCl, 1.5 mM MgCl_2_, 2 mM EDTA, 1% Triton X-100, 0.5 mM DTT, and protease inhibitor cocktail (Sigma-Aldrich). The brain tissue lysates were incubated with the CK2α or Rac1 antibody overnight, then coupled to the immobilized protein A/G agarose beads (Beyotime Institute of Biotechnology, Haimen, China) at 4°C for 4 h. After extensive washing for three times in lysis buffer (a 4-fold lower concentration of NaCl than the original lysis buffer), coimmunoprecipitated Rac1 or CK2α was detected by Western blot with Rac1 or CK2α antibodies. Normal IgG was purchased from Santa Cruz Biotechnology using as a negative control.

### Immunofluorescent staining

At 3 d after reperfusion, the rats were perfused with heparinized (10 U/ml) saline and then with 4% formaldehyde in phosphate buffered salin (PBS). The brain tissues were sectioned at 50 μm using a vibratome and stored at −20°C. The sections were washed three times with PBS, blocked with 5% bovine serum albumin (0.3% Triton X-100 in PBS) for 1 h and incubated overnight in primary antibodies. The primary antibodies used in this experiment were rabbit anti-CK2α (1:100; Millipore) and mouse anti-Rac1 (1:100; Millipore). The sections were washed three times with PBS and then incubated for 60 min with Alexa Fluor 488-conjugated goat anti-mouse IgG and Alexa Fluor 594-conjugated goat anti-rabbit IgG. Sections were rinsed three times with PBS, mounted on glass slides and covered with mounting medium containing DAPI (Beyotime Institute of Biotechnology, Haimen, China).

### Rac1 activation assay

At 3 d after reperfusion, brains were isolated and lysed with Mg^2+^ lysis buffer according to the manufacturer's instruction (Millipore). Briefly, brian tissue lysates (1 mg/500 μl) were incubated with a Rac/cdc42 assay reagent (PAK1-PBD, agarose) at 4°C for 1 h. The pellets were collected by centrifugation at 14,000 *g* for 5 s, washed in lysis buffer, and resuspended in 40 μl of 2× Laemmli buffer. Samples were incubated for 15 min at 30°C with GTPγS as a positive control, while guanosine diphosphate as a negative control. Finally, co-IP was performed with Rac/cdc42 assay reagent (PAK1-PBD, agarose).

### Primary cortical neuron culture

Primary cortical neurons were prepared from E17 SD rats and the cortices were isolated and cultured as described by Ling et al., 2015 [31].

### Oxygen-glucose deprivation and recovery (OGD/R) treatment

The OGD/R treatment was conducted according to our previous studies [31].

### Cell viability assessment

At 24 h post-OGD, neuronal cell death was measured by the MTT assay and lactate dehydrogenase (LDH) activity in the medium was determined in accordance with our previous studies [31].

### Transient transfection of small RNA interference (siRNA)

The transient transfection of siRNA was performed as described by Ling et al., 2015 [31]. The siRNA construct used was obtained as mismatched siRNA control (Negative Control, Santa Cruz Biotechnology, Santa Cruz, CA) and siRNA against CK2 (siCK2, Santa Cruz Biotechnology, Santa Cruz, CA).

### Western blot analysis

The western blot analysis was conducted according to our previous studies [31].

### Statistical analysis

All data are expressed as mean ± SD. Statistical analyses were performed with one-way ANOVA followed by Turkey's *post hoc test* for multiple comparison tests. Significant differences were accepted when *p* < 0.05.

## SUPPLEMENTARY MATERIAL FIGURE


