# Supplementary material for: 5d, a novel analogue of 3-n-butylphthalide, decreases NADPH oxidase activity through the positive regulation of CK2 after ischemia/reperfusion injury
Source: Oncotarget. 2016 May 12;7(26):39444–57. doi: 10.18632/oncotarget.8548 (PMC5129944; doi:10.18632/oncotarget.8548)
Supplement: Supplementary file 1 [file oncotarget-07-39444-s001.pdf]

5d, a novel analogue of 3-n-butylphthalide, decreases NADPH oxidase activity through the positive regulation of CK2 after ischemia/reperfusion injury

Supplementary Material

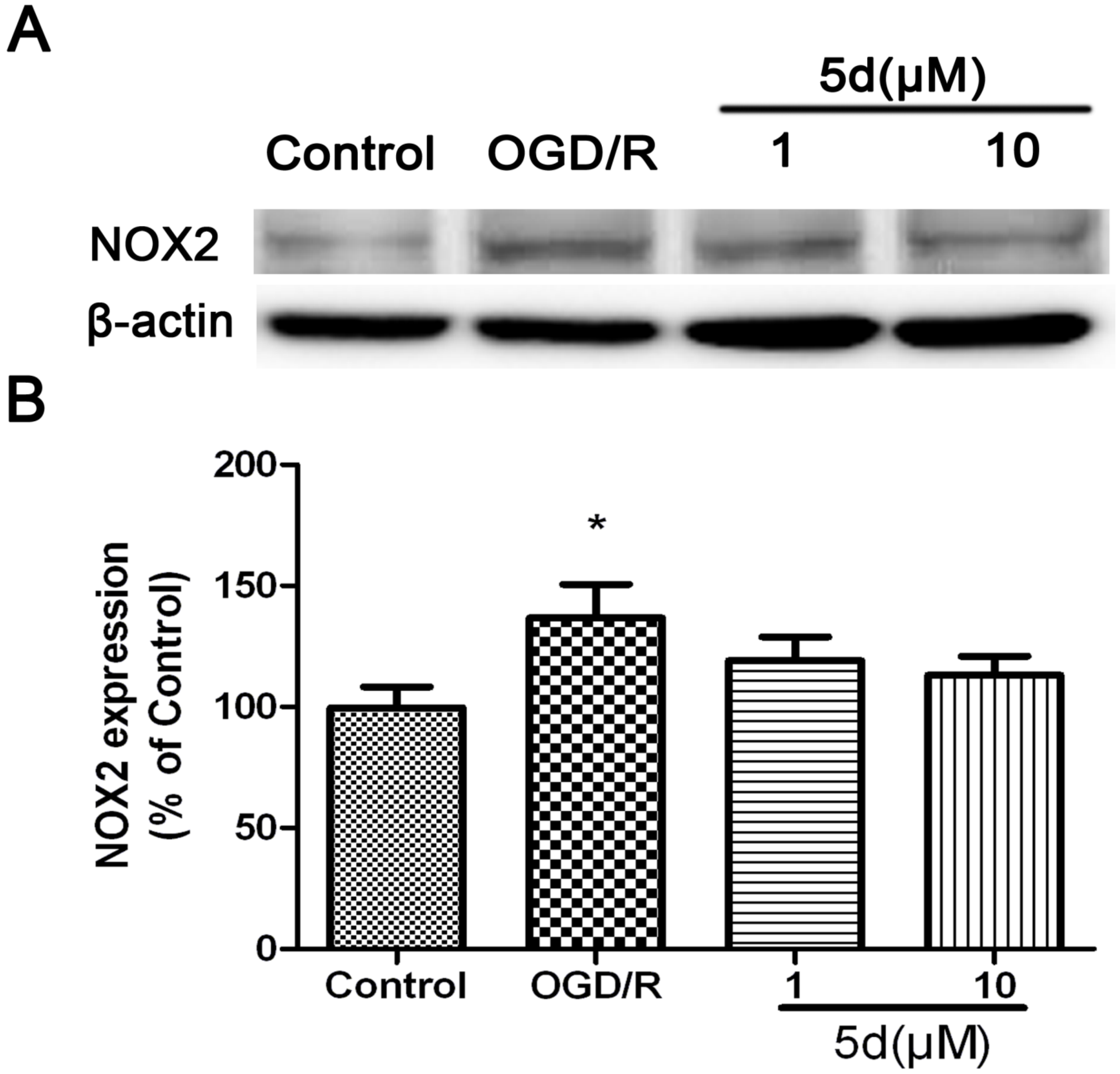

Supplemental Fig.S1 Effects of **5d** on NOX2 protein expression in cortical neurons after OGD/R. (A) Representative Western blots of NOX2 protein expression in cortical neurons. (B) NOX2 protein expression were normalized to β-actin level. Data are expressed as means ± SD (n=4). $^{*}P < 0.05$ *vs* Control group.
